# Secondary acute lymphoblastic leukemia is a distinct clinical entity with prognostic significance

**DOI:** 10.1038/bcj.2017.81

**Published:** 2017-09-08

**Authors:** A S Rosenberg, A Brunson, J K Paulus, J Tuscano, T Wun, T H M Keegan, B A Jonas

**Affiliations:** 1 Center for Oncology Hematology Outcomes Research and Training (COHORT), Division of Hematology Oncology, University of California Davis School of Medicine, Sacramento, CA, USA; 2 University of California Davis Comprehensive Cancer Center, Sacramento, CA, USA; 3 Predictive Analytics and Comparative Effectiveness (PACE) Center, Institute for Clinical Research and Health Policy Studies, Tufts Medical Center, Boston, MA, USA; 4 VA Northern California Health Care System, Sacramento, CA, USA

## Abstract

The effect of prior malignancy on the risk of developing, and prognosis of, acute lymphoblastic leukemia (ALL) is unknown. This observational study utilized the California Cancer Registry to estimate the risk of developing ALL after a prior malignancy using standardized incidence ratios (SIRs, 95% confidence intervals). ALL occurring after a malignancy with an SIR>1 (increased-risk (IR) malignancies) was considered secondary ALL (s-ALL). Adjusted hazard ratios (aHRs, 95% confidence intervals) compared the effect of s-ALL with *de novo* ALL on overall survival. A total of 14 481 patients with ALL were identified (1988–2012) and 382 (3%) had a known prior malignancy. Any prior malignancy predisposed patients to developing ALL: SIR 1.62 (1.45–1.79). Hematologic malignancies (SIR 5.57, 4.38–6.98) and IR-solid tumors (SIR 2.11, 1.73–2.54) increased the risk of developing ALL. s-ALL increased the risk of death compared with *de novo* ALL (aHR 1.38 (1.16–1.63)) and this effect was more pronounced among younger patients (age<40 years: aHR 4.80 (3.15–7.30); age⩾40 years: aHR 1.40 (1.16–1.69)) (interaction *P*<0.001). This population-based study demonstrates that s-ALL is a distinct entity that occurs after specific malignancies and carries a poor prognosis compared with *de novo* ALL, particularly among patients <40 years of age.

## Introduction

As cancer survival continues to improve, second primary malignancies are becoming an increasingly common problem facing both clinicians and cancer survivors. Acute myelogenous leukemia evolving from an underlying bone marrow disorder (secondary acute myelogenous leukemia) or as a result of radiation or chemotherapy (therapy-related acute myelogenous leukemia) is well described.^1, 2, 3^ By comparison, little is known about acute lymphoblastic leukemia (ALL) occurring after an antecedent malignancy (am-ALL). One large analysis of the Surveillance, Epidemiology and End Results (SEER) cancer registry program found that the incidence of ALL was higher than expected in survivors of Hodgkin lymphoma, small cell lung and ovarian cancers, and that younger patients were nearly 20 times more likely to develop ALL than the background population.^4^ Five single institution case series,^5, 6, 7, 8, 9^ one leukemia-specific registry,^10^ four summaries of prior case series^11, 12, 13, 14^ and two SEER-based studies^15, 16^ have examined ALL patients for a history of prior malignancy and treatment, and identified prior malignancies among <1% to 9.6% of ALL patients. Rearrangements of the MLL gene on chromosome 11 appear more common among ALL patients with prior malignancies compared with *de novo* ALL, supporting the argument that secondary ALL (s-ALL) is a distinct entity, and may be linked to specific therapies.^5, 6, 7, 14^ These s-ALL cases with MLL and other cytogenetic alterations have been labeled therapy-related ALL (t-ALL) in many of these studies.^6, 7, 9, 12, 13^ This line of reasoning has been challenged, however, by the observed high rates of malignancies within families of ALL patients with a prior malignancy, leading some to posit that s-ALL is instead due to constitutional susceptibility to malignancy.^8, 10, 14^

Although studies have described frequencies of prior malignancies among ALL patients,^5, 6, 7, 8, 10, 11^ no prior study has focused on the risk of developing ALL among cancer survivors. Furthermore, the prognostic impact of a prior malignancy on ALL patients is uncertain.^1, 2, 15, 17^ Therefore, we utilized data from the population-based California Cancer Registry (CCR) to determine whether ALL rates are higher among cancer survivors with specific tumors and whether ALL in this context confers a worse prognosis. Findings from this study will have important implications for patients followed in survivorship clinics and for risk stratification and treatment of ALL.

## Materials and methods

Patients were identified using the CCR database. California state law mandates that all cancers diagnosed in California are reported to the CCR since 1 January 1988.^18, 19, 20^ The CCR abstracts high-quality data from medical records including demographic characteristics (race/ethnicity, gender, age at diagnosis, marital status, health insurance and neighborhood socioeconomic status), tumor characteristics (primary cancer site, tumor sequence and stage at diagnosis), initial course of treatment (chemotherapy, radiation and surgery), vital status and follow-up time. Vital status and follow-up time were complete through 31 December 2012. We developed a conceptual framework before analysis that included am-ALL as a subset of *de novo* ALL, and that among am-ALL patients some would have s-ALL and/or t-ALL (Supplementary Figure 1).

### Patient identification

All patients diagnosed with ALL from 1 January 1988 to 31 December 2012 were identified using the SEER site recode for ALL (35 011).^21^ Tumor sequence was used to identify first primary ALL (00 or 01) or am-ALL (>01). To ensure the am-ALL diagnosis was a subsequent primary malignancy, patients with a first primary acute leukemia, chronic myelogenous leukemia, Burkitt, or lymphoblastic lymphoma or unknown first primary were excluded from the analyses. Patients with prior chronic lymphocytic leukemia, chronic myelo-monocytic leukemia and chronic eosinophilic leukemia were included (*n*=12). Patients with a latency period of less than 60 days between their first primary cancer and am-ALL were excluded.^22^

### Statistical analysis

Categorical data was analyzed using *χ*^2^-testing and two-sided *P*-values <0.05 were considered statistically significant. Standardized incidence ratios (SIRs), defined as the ratio of observed number of ALL cases among cancer survivors to the expected number of *de novo* ALL in the general population of California, and corresponding 95% confidence intervals were calculated to estimate the risk of am-ALL. SEER site recode was utilized to classify types of cancers.^23^ Expected numbers of *de novo* ALL were calculated by gender, race/ethnicity, 5-year age-categories and 3-year time periods using first primary ALL rates from the CCR. SIRs were calculated in strata defined by cancer type and age category, pediatric/adolescent and young adult (pediatric/AYA, age<40 years) and older adults (age⩾40 years), based on the higher risks of subsequent cancers in younger compared with older patients.^24^ Malignancies with statistically significant SIRs >1 were considered ‘increased risk (IR)’ and ALL developing after an IR primary was considered s-ALL, as compared with ALL developing after any am-ALL.

Multivariable cox proportional hazard models were used to compare survival of am-ALL compared with *de novo* ALL adjusting for characteristics at diagnosis, including age, sex, race/ethnicity, marital status, health insurance, urban residence, neighborhood socioeconomic status and year of diagnosis. Two primary outcome models included overall survival of (1) am-ALL vs *de novo* ALL and (2) s-ALL vs *de novo* ALL. For deceased patients, survival time was measured in days from the date of diagnosis to the date of death from any cause. Patients alive at the study end date (31 December 2012) were censored at this time or at the date of last known contact. Cross-product interactions of am-ALL and s-ALL with age group were evaluated in all overall survival models. In all models, the proportional hazards assumption was assessed using Schoenfeld residuals.^25^

The CCR records planned first course of treatment including whether chemotherapy or radiation therapy was employed. Data on specific types of chemotherapy, doses of chemotherapy or radiation therapy, and areas of involved treatment are not available in this data set. Therefore, an exploratory analysis on the effects of initial prior therapy on both the development, via SIR analysis, and survival, via Cox models, of am-ALL were performed.

SIRs were calculated in SEER*Stat version 8.3.2 (National Cancer Institute, Bethesda, MD, USA), all other analysis was performed using SAS Version 9.4 (SAS Institute Inc., Cary, NC, USA). This study was approved by the Committee for the Protection of Human Subjects of the California and the University of California, Davis Institutional Review boards.

## Results

### Patient characteristics

A total of 14 470 patients with ALL were identified. Of these, 14 099 (97%) had *de novo* ALL and 371 (3%) had am-ALL (Table 1). Differences in sex, race/ethnicity and neighborhood socioeconomic status were noted between am-ALL and *de novo* ALL. Pediatric/AYA cases comprised 78% (*n*=11 229) of primary ALL cases, but these cases represented only 12% (*n*=43) of the am-ALL cohort. The most common prior malignancies were breast cancer (21%), hematologic malignancies (18%) and male genital system (15%).

### Risk of ALL among cancer survivors

**Figure 1:**
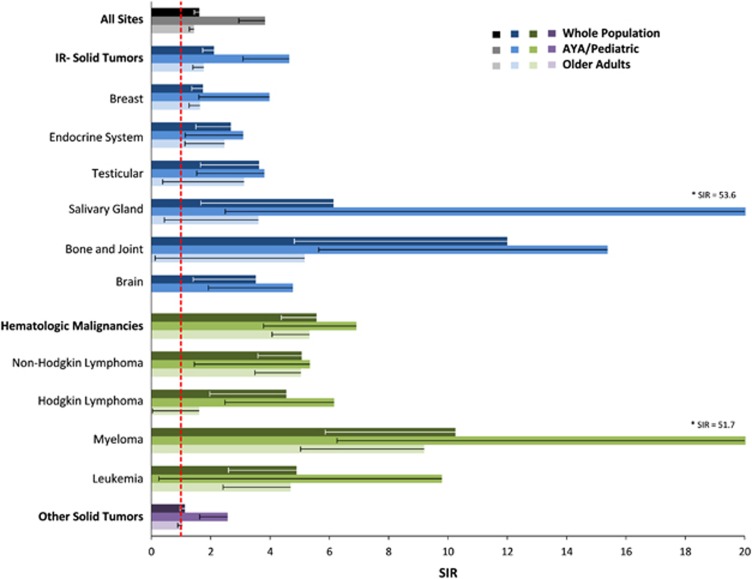
SIRs for acute lymphoblastic leukemia preceded by prior malignancy. IR solid tumors defined as those solid tumors with SIRs >1. Older adults are defined as age ⩾40 years and pediatric/AYA are defined as age <40 years. Lower 95% confidence intervals are noted by the whisker.

Compared with the background population of California, cancer survivors had a 62% increase in the incidence of ALL (SIR 1.62 (1.45–1.79)). Hematologic malignancies conferred more than five-fold IR (SIR 5.57, (4.38–6.98)) and solid tumors conferred a 37% IR (SIR 1.37 (1.21, 1.53)); however, this risk was driven by specific ‘IR’ solid tumors (Figure 1 and Supplementary Table 1). Although male and female genitourinary, colorectal and skin cancers were common antecedent malignancies due to their prevalence in the background population, they were not associated with IR of developing ALL. Based on these findings, ALL arising after any IR malignancy, hematologic or solid was considered s-ALL in subsequent analyses.

The effect of prior malignancies on the development of s-ALL was greater in pediatric/AYA than older patients (Figure 1). Notably, IR solid tumors conferred IR in both age categories, but the risk was higher among pediatric/AYA (SIR 4.65 (3.09–6.72)) than older (SIR 1.77 (1.40–2.20)) patients. Conversely, among non-IR solid tumors, the risk of developing ALL was not increased among older adults (SIR 1.04 (0.89–1.22)), but was over 2.5-fold higher (SIR 2.57 (1.63–3.86)) in pediatric/AYA patients. This effect was driven primarily by rectal cancers (*n*=3; SIR 22.56 (4.65–65.93)), which remained non-IR for the entire cohort (*n*=9; SIR 1.87 (0.86–3.55)).

**Figure 2:**
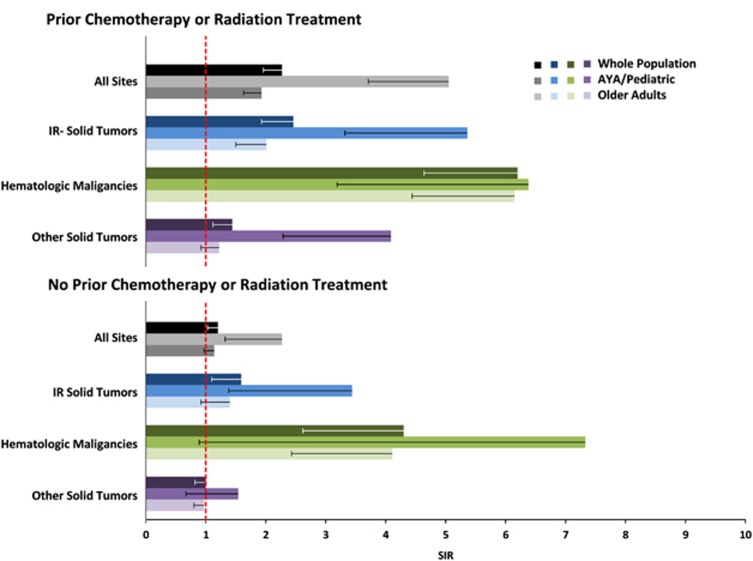
SIRs for acute lymphoblastic leukemia preceded by prior chemotherapy/radiation therapy. IR solid tumors defined as those solid tumors with SIRs >1. Prior treatment refers treatment with chemotherapy and/or radiation therapy for the prior malignancy. Older adults are defined as age ⩾40 years and pediatric/AYA are defined as age <40 years. Lower 95% confidence intervals are noted by the whisker.

Among all cancer survivors, those with prior cancer treatment had more than twice the incidence of ALL (SIR 2.27 (1.96–2.61)) in both pediatric/AYA (SIR 5.05 (3.71–6.71)) and older adults (1.93 (1.63–2.26)) (Figure 2). Those with no prior treatment had a small, but significant increase in ALL incidence (SIR 1.20 (1.03–1.40)) that was driven by pediatric/AYA patients (SIR 2.27 (1.32–3.63)).

### Time from first primary to ALL

The median time from primary malignancy to am-ALL was 67 months (range 2.6–277). The median time from hematologic malignancy was 68 months (2.6–221) and from IR solid tumors was 69 months (3.8–277). The time from non-IR solid tumors to am-ALL did not differ greatly (median 64 months (3–269)) (data not shown).

### Survival of secondary ALL

To compare the overall survival of *de novo* ALL and am-ALL, Cox proportional models were utilized that accounted for age at diagnosis, gender, race/ethnicity, era of diagnosis (to account for changes in treatment and outcome over time) and socioeconomic status. The hazard of death was increased in am-ALL patients compared with *de novo* ALL (adjusted hazard ratio (aHR) 1.19 (1.05–1.34)) (Table 2). However, when taking into account the type of prior malignancy, the hazard of death remained elevated for s-ALL patients when compared with *de novo* ALL (aHR: 1.38 (1.16–1.63)), whereas there was no difference in the hazard of death between patients with non-IR solid primary cancers and *de novo* ALL. The effect of prior IR solid tumors and prior hematologic malignancy on survival compared with *de novo* ALL did not differ significantly (aHR 1.54 (1.16–2.03) and 1.30 (1.05–1.61), respectively, *P* for interaction=0.3449) (data not shown) and therefore s-ALL was analyzed as a single group.

The effect of s-ALL on survival differed by age (*P* for interaction <0.001), with the effect of s-ALL being worse in pediatric/AYA patients compared with older adults (aHR 4.80 (3.15–7.30) and 1.40 (1.16–1.69), respectively) (Table 2). Unlike pediatric/AYA patients, older adults with a non-IR prior malignancy also experienced an increased hazard of death.

In exploratory analysis, cancer survivors with am-ALL who received either chemotherapy or radiation for their prior cancer were at significantly IR of death compared to *de novo* ALL patients (aHR 1.26 (1.06–1.50)), whereas cancer survivors with am-ALL, who did not receive chemotherapy or radiation for their prior cancer, had similar survival to *de novo* ALL patients (aHR 1.13 (0.96–1.33)) (data not shown).

## Discussion

Over the last two decades, several reports on the development of ALL as a second malignancy have attempted to identify patterns that could link either prior disease states or prior therapy and ALL development. To our knowledge, the current study represents the largest population-based study of *de novo* compared with am-ALL to date and the only study that determined whether a difference exists in the incidence of ALL in cancer survivors compared with the general population. The increased SIR for patients with IR malignancies argues for a link between either therapy^6^ or an underlying constitutional predisposition^8, 10^ shared by those malignancies and ALL.

In the current study, we found that ALL occurs at an increased rate among a specific subset of solid tumors and hematologic malignancies. Among pediatric/AYA patients, the effect of ALL arising as a second malignancy on survival differs depending on whether it arose after a IR malignancy or not, with a significantly worse prognosis for s-ALL compared with *de novo* ALL. In contrast, in older adults any prior malignancy was associated with an IR of death. A number of factors may contribute to this latter finding, including older adult ALL patients having poor baseline survival rates, making a change in relative survival more difficult to detect, being less likely to tolerate intensive chemotherapy after having been treated for any prior malignancy or having more comorbidities especially among cancer survivors. The current study could not evaluate stem cell transplant utilization or its effect on survival. Different transplant utilization rates in *de novo* vs s-ALL among pediatric/AYA and adult populations may account for some of the difference observed in the current study and will be the subject of a future analysis. Taken together, the IR after specific malignancies coupled with differences in survival, especially among pediatric/AYA patients, supports the hypothesis that s-ALL is a distinct entity with prognostic relevance.

The effect of prior malignancy on the survival of ALL has been reported variously, with three case series published since 2010 reporting median survival times of about 1 year.^6, 7, 8^ Only one of the reports compared survival of am-ALL with *de* novo ALL, with median OS of 12 months compared with 45 months, with a median follow-up of 24 months. After accounting for baseline characteristics however, prior malignancy had no effect on survival. Another report focusing on am-ALL patients undergoing stem cell transplant found a 4-year overall survival rate of 51%.^9^ Whether this apparent difference in survival is due to the ability to undergo stem cell transplant or the effect of the treatment remains unexplored in the literature. Recently, two large SEER studies evaluated the prognostic impact of s-ALL and it was associated with an inferior overall survival compared with *de novo* ALL.^15, 16^ However, these studies did not include children<18 or consider interactions by age, used a pre-defined subset of prior malignancies rather than considering all prior malignancies and did not consider prior therapy in their analyses. Importantly, these studies did not report SIRs for the prior malignancies used in their analyses and it is not clear whether they considered the possibility that some am-ALL cases may reflect *de novo* ALL.

Aberrations in the MLL gene, most commonly t(4;11),^5, 6, 7, 8, 9, 10^ hypodiploidy^6^ and the presence of t(9;22)^7, 8, 9, 10^ are frequent findings in am-ALL. In two reports comparing patient characteristics of am-ALL patients who had, or had not, received prior chemotherapy or radiation, 88% (*n*=7)(6) and 100% (*n*=2)(8) of patients with t(4;11) had prior therapy, whereas 44% (*n*=7)(6) and 67% (*n*=8)(8) patients with the t(9;22) had prior therapy, suggesting an association between both cytogenetic defects and prior therapy. Accordingly, these and other studies have concluded that cases of am-ALL following prior chemotherapy/radiation are best characterized as t-ALL. In our exploratory analysis considering treatment of a prior malignancy, both chemotherapy and radiation were associated with a higher incidence of ALL among cancer survivors and higher risk of death after am-ALL. The differences between the SIRs in our primary analysis and the exploratory analysis considering prior treatment suggest distinct s-ALL and t-ALL subpopulations probably exist in addition to an overlapping subpopulation of s-ALL and t-ALL. Taken in sum, the current study supports the hypothesis that am-ALL is a heterogeneous subset of ALL comprising s-ALL, which occurs as a result of an underlying predisposition, either constitutional or disease-related,^8, 10^ t-ALL, which occurs as a result of chemotherapy/radiation in IR and some non-IR prior malignancies, and ALL arising after non-IR malignancies, which may be more akin to *de novo* ALL.

This study of the CCR has limitations. It does not contain cytogenetic or molecular data. The survival analyses may be subject to index event bias.^26^ Although this type of bias is unpredictable in its direction and effect, we anticipate it would bias the survival analysis away from statistical significance. Treatment data in the CCR likely under-ascertains full-course of therapy. Thus, analyses based on treatment were considered exploratory and should be interpreted with some caution, as it is likely that a proportion of patients recorded as not receiving initial treatment, in fact did. However, under-ascertainment of treatment is more likely to bias our results towards the conclusion that there is no difference in risk of developing ALL or in survival between those patients receiving prior treatment and those who did not. Therefore, both the SIR and survival data presented is likely to underestimate the true effect of IR-malignancies on the development of s-ALL and of s-ALL on survival. Taken in sum, we present evidence for s-ALL and t-ALL in agreement with prior reports.^6, 7^

There are also a number of strengths in the population-based data source. The CCR includes >99% of all invasive cancers diagnosed in the state of California. California has a large, socioeconomically and racial/ethnically diverse population, and includes patients treated in academic and community settings, thus making these results more generalizable than prior studies. This is the first study to comprehensively consider the effect of all prior malignancies on the incidence of ALL, and the largest study, to date, to compare overall survival of patients with *de novo* and s-ALL.

In conclusion, s-ALL is a distinct entity from *de novo* ALL and carries adverse prognostic implications that vary by age. Secondary ALL is of particular relevance in the pediatric/AYA population, in whom ALL would be otherwise quite treatable with a high rate of cure. Furthermore, the existence of t-ALL as a distinct subtype of ALL, sharing overlap with s-ALL, is suggested by this study. Further exploration of the potential relationship with specific chemotherapeutic and radiation regimens is both warranted and necessary to understand the genetic and molecular underpinnings s-ALL.

## Figures and Tables

**Table 1:** Patient characteristicsAbbreviations: ALL, acute lymphoblastic leukemia; am-ALL, antecedent malignancy ALL.

*Patient characteristics*	*All*		De novo *ALL*		*am-ALL*		P*-value*
	N	*%*	N	*%*	N	*%*	
All	14 470	100.0%	14 099	100.0%	371	100.0%	
							
*Gender*							
Male	8191	56.6%	8018	56.9%	173	46.6%	<0.0001
Female	6279	43.4%	6081	43.1%	198	53.4%	<0.0001
							
*Race/ethnicity*							
Non-Hispanic White	5495	38.0%	5267	37.4%	228	61.5%	<0.0001
African American	558	3.9%	542	3.8%	16	4.3%	00.6437
Hispanic	6985	48.3%	6889	48.9%	96	25.9%	<0.0001
Asian/Pacific Islander	1280	8.8%	1249	8.9%	31	8.4%	0.7363
Other/unknown	152	1.1%	152	1.1%			0.0444
							
*Age at diagnosis*							
Age 0–9	6752	46.7%	6746	47.8%	6	1.6%	<0.0001
10–19	2663	18.4%	2648	18.8%	15	4.0%	<0.0001
20–29	935	6.5%	932	6.6%	3	0.8%	<0.0001
30–39	879	6.1%	860	6.1%	19	5.1%	0.4361
40–49	837	5.8%	806	5.7%	31	8.4%	0.0316
50–59	778	5.4%	715	5.1%	63	17.0%	<0.0001
60–69	669	4.6%	590	4.2%	79	21.3%	<0.0001
70–79	561	3.9%	473	3.4%	88	23.7%	<0.0001
80+	396	2.7%	329	2.3%	67	18.1%	<0.0001
							
*Year of diagnosis*							
1988–1992	2315	16.0%	2301	16.3%	14	3.8%	<0.0001
1993–1997	2647	18.3%	2605	18.5%	42	11.3%	0.0004
1998–2002	2838	19.6%	2779	19.7%	59	15.9%	0.0683
2003–2007	3186	22.0%	3078	21.8%	108	29.1%	0.0008
2008–2012	3484	24.1%	3336	23.7%	148	39.9%	<0.0001
							
*ALL histology*							
Precursor cell, B cell	5208	36.0%	5018	35.6%	190	51.2%	<0.0001
Precursor cell, NOS	8701	60.1%	8535	60.5%	166	44.7%	<0.0001
Precursor cell, T cell	561	3.9%	546	3.9%	15	4.0%	0.8666
							
*Neighborhood socioeconomic status*							
Low	9528	65.8%	9347	66.3%	181	48.8%	<0.0001
High	4786	33.1%	4604	32.7%	182	49.1%	<0.0001
Unknown	156	1.1%	148	1.0%	8	2.2%	0.0416
							
*Previous treatment*							
Chemo or radiation	184	1.3%			184	49.6%	
None	14 286	98.7%			187	50.4%	
							
*First known tumor*							
No previous tumor	14099	97.4%	14099	100.0%			
Oral cavity/pharynx	7	0.0%			7	1.9%	
Digestive system	37	0.3%			37	10.0%	
Respiratory system	14	0.1%			14	3.8%	
Bone/joints	6	0.0%			6	1.6%	
Soft tissue (inc heart)	5	0.0%			5	1.3%	
Skin	27	0.2%			27	7.3%	
Breast	77	0.5%			77	20.8%	
Female genital system	25	0.2%			25	6.7%	
Male genital system	57	0.4%			57	15.4%	
Urinary system	14	0.1%			14	3.8%	
Eye/orbit	2	0.0%			2	0.5%	
Brain/other nervous system	7	0.0%			7	1.9%	
Endocrine system	15	0.1%			15	4.0%	
Lymphoma	40	0.3%			40	10.8%	
Myeloma	13	0.1%			13	3.5%	
Leukemia	12	0.1%			12	3.2%	
Mesothelioma	1	0.0%			1	0.3%	
Kaposi sarcoma	2	0.0%			2	0.5%	
Misc	10	0.1%			10	2.7%	

**Table 2:** Effect of prior malignancy on hazard of death from all causes among ALL patients by age group, California, 1988–2012

*Model*	*Variable*	*aHR*	*95% CI*	P*-value*
A	*de novo* ALL	REF		
	am-ALL^a^	1.19	(1.05, 1.34)	0.0062
				
B	*de novo* ALL	REF		
	s-ALL^b^	1.38	(1.16, 1.63)	0.0003
	Prior non-IR solid tumors^c^	1.05	(0.89, 1.24)	0.5536
				
C	s-ALL^b^ (vs *de novo* ALL)			
	Age<40 years	4.80	(3.15, 7.30)	<.0001
	Age⩾40 years	1.40	(1.16, 1.69)	0.0004
	Prior non-IR solid tumors^c^ (vs *de novo* ALL)			
	Age<40 years	1.34	(0.34, 5.37)	0.6781
	Age⩾40 years	1.54	(1.31, 1.81)	<.0001

Abbreviations: CI, confidence interval; aHR, adjusted hazard ratio of death; ALL, acute lymphoblastic leukemia; am-ALL, antecedent malignancy ALL; IR, increased risk; s-ALL, secondary ALL.

All models accounting for sex, race/ethnicity, age at diagnosis, marital status, year of diagnosis, histology (B-cell, T-cell and not-otherwise-specified), neighborhood socioeconomic status, rural (vs urban) location.

a am-ALL is defined as ALL after any prior malignancy.

b s-ALL is defined as ALL occurring after IR malignancies (hematologic malignancies and salivary gland, bone and joint, breast, testis, brain, thyroid and other endocrine including thymus cancers).

c Non-IR solid tumors were all malignancies not associated with an increased risk of developing ALL as a second malignancy.
