# Supplementary material for: Secondary acute lymphoblastic leukemia is a distinct clinical entity with prognostic significance
Source: Blood Cancer J. 2017 Sep 8;7(9):e605–. doi: 10.1038/bcj.2017.81 (PMC5709750; doi:10.1038/bcj.2017.81)
Supplement: Supplementary Figure 1 [file bcj201781x2.docx]

**Supplemental Figure 1:** Proposed Framework for Analyzing the Patients with Acute Lymphoblastic Leukemia Identified in the California Cancer Registry

Legend: ALL arising after any antecedent malignancy (am-ALL) comprises a subset of all ALL cases. Within the am-ALL population, there are overlapping subsets of patients in whom some predisposing factor affects the risk of developing ALL (s-ALL) which is associated with specific primary cancers. In addition, prior treatment with chemotherapy and radiation therapy increases the risk of developing ALL (t-ALL), and there is overlap between s-ALL and t-ALL.
