# Supplementary material for: Secondary acute lymphoblastic leukemia is a distinct clinical entity with prognostic significance
Source: Blood Cancer J. 2017 Sep 8;7(9):e605–. doi: 10.1038/bcj.2017.81 (PMC5709750; doi:10.1038/bcj.2017.81)
Supplement: Supplementary file 2 [file bcj201781x1.docx]

Supplemental Table 1: Standardized Incidence Ratio by Prior Cancer Site and Age at Diagnosis, California

Cancer Site	Whole Population				AYA/Pediatric				Older Adults			
	O	E	SIR	95% CI	O	E	SIR	95% CI	O	E	SIR	95% CI
**All Sites**	365	225.92	1.62*	(1.45, 1.79)	65	16.98	3.83*	(2.95, 4.88)	300	208.94	1.44*	(1.28, 1.61)
**Solid Tumors**	290	212.44	1.37*	(1.21, 1.53)	51	14.95	3.41*	(2.54, 4.48)	239	197.49	1.21*	(1.06, 1.37)
IR- Solid	108	51.28	2.11*	(1.73, 2.54)	28	6.02	4.65*	(3.09, 6.72)	80	45.26	1.77*	(1.40, 2.20)
Other Solid	182	161.16	1.13	(0.97, 1.31)	23	8.94	2.57*	(1.63, 3.86)	159	152.23	1.04	(0.89, 1.22)
**Hematologic Malignancies**	75	13.48	5.57*	(4.38, 6.98)	14	2.03	6.91*	(3.78, 11.60)	61	11.45	5.33*	(4.07, 6.84)
**Individual Cancer Sites**												
**Oral Cavity and Pharynx**	10	4.9	2.04	(0.98, 3.75)	3	0.36	8.30*	(1.71, 24.27)	7	4.54	1.54	(0.62, 3.18)
Tongue	2	1.13	1.77	(0.21, 6.38)	-	-	-	-	2	1.08	1.85	(0.22, 6.70)
Salivary Gland	4	0.65	6.14*	(1.67, 15.73)	2	0.1	20.52*	(2.49, 74.13)	2	0.55	3.61	(0.44, 13.05)
Gum and Other Mouth	1	0.65	1.54	(0.04, 8.58)	-	-	-	-	1	0.6	1.67	(0.04, 9.28)
Nasopharynx	1	0.33	3.03	(0.08, 16.90)	1	0.08	12.52	(0.32, 69.77)	-	-	-	-
Tonsil	2	0.67	2.99	(0.36, 10.80)	-	-	-	-	2	0.65	3.07	(0.37, 11.11)
**Digestive System**	33	29.95	1.1	(0.76, 1.55)	3	0.91	3.31	(0.68, 9.67)	30	29.05	1.03	(0.70, 1.47)
Colon and Rectum	29	23.74	1.22	(0.82, 1.75)	3	0.45	6.65*	(1.37, 19.42)	26	23.29	1.12	(0.73, 1.64)
Colon excluding Rectum	17	16.59	1.02	(0.60, 1.64)	-	-	-	-	17	16.31	1.04	(0.61, 1.67)
Cecum	3	3.56	0.84	(0.17, 2.46)	-	-	-	-	3	3.51	0.85	(0.18, 2.49)
Ascending Colon	3	2.59	1.16	(0.24, 3.39)	-	-	-	-	3	2.56	1.17	(0.24, 3.43)
Hepatic Flexure	2	0.89	2.26	(0.27, 8.16)	-	-	-	-	2	0.87	2.3	(0.28, 8.31)
Sigmoid Colon	7	6.01	1.17	(0.47, 2.40)	-	-	-	-	7	5.92	1.18	(0.48, 2.44)
Large Intestine, NOS	2	0.34	5.9	(0.71, 21.31)	-	-	-	-	2	0.33	6.15	(0.74, 22.22)
Rectum and Rectosigmoid Junction	12	7.15	1.68	(0.87, 2.93)	3	0.17	17.28*	(3.56, 50.51)	9	6.98	1.29	(0.59, 2.45)
Rectosigmoid Junction	3	2.34	1.28	(0.26, 3.74)	-	-	-	-	3	2.3	1.3	(0.27, 3.81)
Rectum	9	4.81	1.87	(0.86, 3.55)	3	0.13	22.56*	(4.65, 65.93)	6	4.68	1.28	(0.47, 2.79)
Anus, Anal Canal and	1	0.71	1.42	(0.04, 7.89)	-	-	-	-	1	0.68	1.48	(0.04, 8.23)
Liver and Intrahepatic Bile Duct	2	0.96	2.09	(0.25, 7.55)	-	-	-	-	2	0.78	2.57	(0.31, 9.27)
Liver	2	0.9	2.22	(0.27, 8.02)	-	-	-	-	2	0.72	2.76	(0.33, 9.98)
Pancreas	1	0.71	1.41	(0.04, 7.83)	-	-	-	-	1	0.68	1.47	(0.04, 8.18)
**Respiratory System**	15	10.15	1.48	(0.83, 2.44)	1	0.28	3.6	(0.09, 20.06)	14	9.87	1.42	(0.78, 2.38)
Larynx	3	1.89	1.59	(0.33, 4.64)	-	-	-	-	3	1.87	1.61	(0.33, 4.70)
Lung and Bronchus	11	7.82	1.41	(0.70, 2.52)	-	-	-	-	11	7.71	1.43	(0.71, 2.55)
Trachea, Mediastinum and Other Respiratory Organs	1	0.13	7.71	(0.20, 42.96)	1	0.1	9.66	(0.24, 53.84)	-	-	-	-
**Bones and Joints**	7	0.58	12.00*	(4.82, 24.72)	6	0.39	15.38*	(5.64, 33.48)	1	0.19	5.17	(0.13, 28.80)
**Soft Tissue including Heart**	5	1.86	2.69	(0.88, 6.29)	3	0.77	3.9	(0.80, 11.40)	2	1.09	1.84	(0.22, 6.65)
**Skin excluding Basal and**	18	12.12	1.49	(0.88, 2.35)	2	1.69	1.18	(0.14, 4.26)	16	10.42	1.53	(0.88, 2.49)
Melanoma of the Skin	16	10.97	1.46	(0.83, 2.37)	2	1.51	1.32	(0.16, 4.78)	14	9.46	1.48	(0.81, 2.48)
Other Non-Epithelial Skin	2	1.15	1.74	(0.21, 6.29)	-	-	-	-	2	0.97	2.07	(0.25, 7.48)
**Breast**	73	41.97	1.74*	(1.36, 2.19)	7	1.76	3.98*	(1.60, 8.20)	66	40.21	1.64*	(1.27, 2.09)
**Female Genital System**	19	14.19	1.34	(0.81, 2.09)	1	1.49	0.67	(0.02, 3.74)	18	12.7	1.42	(0.84, 2.24)
Cervix Uteri	5	3.01	1.66	(0.54, 3.88)	-	-	-	-	5	2.18	2.29	(0.74, 5.35)
Corpus and Uterus, NOS	10	7.97	1.26	(0.60, 2.31)	-	-	-	-	10	7.72	1.3	(0.62, 2.38)
Corpus Uteri	10	7.92	1.26	(0.61, 2.32)	-	-	-	-	10	7.68	1.3	(0.62, 2.40)
Ovary	3	2.45	1.22	(0.25, 3.58)	1	0.34	2.96	(0.08, 16.51)	2	2.11	0.95	(0.11, 3.42)
Vagina	1	0.12	8.06	(0.20, 44.91)	-	-	-	-	1	0.11	9.43	(0.24, 52.55)
**Male Genital System**	65	67.41	0.96	(0.74, 1.23)	7	1.88	3.72*	(1.50, 7.66)	58	65.53	0.89	(0.67, 1.14)
Prostate	56	64.63	0.87	(0.65, 1.13)	-	-	-	-	56	64.61	0.87	(0.65, 1.13)
Testis	9	2.48	3.63*	(1.66, 6.89)	7	1.84	3.81*	(1.53, 7.85)	2	0.64	3.12	(0.38, 11.25)
**Urinary System**	17	18.26	0.93	(0.54, 1.49)	3	0.89	3.35	(0.69, 9.80)	14	17.36	0.81	(0.44, 1.35)
Urinary Bladder	12	12.18	0.99	(0.51, 1.72)	-	-	-	-	12	11.99	1	(0.52, 1.75)
Kidney and Renal Pelvis	5	5.8	0.86	(0.28, 2.01)	3	0.7	4.27	(0.88, 12.49)	2	5.1	0.39	(0.05, 1.42)
**Eye and Orbit**	1	0.91	1.1	(0.03, 6.10)	1	0.46	2.16	(0.05, 12.05)	-	-	-	-
**Brain and Other Nervous System**	7	2.33	3.01*	(1.21, 6.20)	7	1.68	4.18*	(1.68, 8.61)	-	-	-	-
Brain	7	1.99	3.52*	(1.41, 7.25)	7	1.47	4.77*	(1.92, 9.84)	-	-	-	-
**Endocrine System**	15	5.59	2.68*	(1.50, 4.42)	6	1.94	3.10*	(1.14, 6.75)	9	3.66	2.46*	(1.13, 4.67)
Thyroid	11	5.06	2.17*	(1.08, 3.89)	3	1.58	1.9	(0.39, 5.55)	8	3.49	2.3	(0.99, 4.52)
Other Endocrine including	4	0.53	7.57*	(2.06, 19.38)	3	0.36	8.40*	(1.73, 24.56)	1	0.17	5.84	(0.15, 32.52)
**Lymphoma**	46	9.26	4.97*	(3.64, 6.63)	11	1.88	5.84*	(2.91, 10.44)	35	7.37	4.75*	(3.31, 6.60)
Hodgkin Lymphoma	8	1.76	4.55*	(1.97, 8.97)	7	1.14	6.16*	(2.48, 12.70)	1	0.62	1.61	(0.04, 8.97)
Hodgkin - Nodal	8	1.73	4.63*	(2.00, 9.13)	7	1.12	6.24*	(2.51, 12.85)	1	0.6	1.66	(0.04, 9.23)
Non-Hodgkin Lymphoma	38	7.5	5.07*	(3.59, 6.95)	4	0.75	5.34*	(1.45, 13.67)	34	6.75	5.04*	(3.49, 7.04)
NHL - Nodal	26	4.85	5.37*	(3.50, 7.86)	3	0.47	6.36*	(1.31, 18.59)	23	4.37	5.26*	(3.33, 7.89)
NHL - Extranodal	12	2.65	4.52*	(2.34, 7.90)	1	0.28	3.6	(0.09, 20.07)	11	2.38	4.63*	(2.31, 8.28)
**Myeloma**	16	1.56	10.25*	(5.86, 16.65)	2	0.04	51.69*	(6.26, 186.72)	14	1.52	9.20*	(5.03, 15.44)
**Leukemia**	13	2.66	4.89*	(2.60, 8.36)	1	0.1	9.8	(0.25, 54.58)	12	2.56	4.69*	(2.42, 8.19)
Lymphocytic Leukemia	10	2.49	4.02*	(1.93, 7.40)	-	-	-	-	10	2.44	4.09*	(1.96, 7.52)
Chronic Lymphocytic Leukemia	10	2.22	4.51*	(2.16, 8.28)	-	-	-	-	10	2.2	4.54*	(2.18, 8.36)
Myeloid and Monocytic Leukemia	1	0.12	8.44	(0.21, 47.04)	-	-	-	-	1	0.07	13.98	(0.35, 77.90)
Chronic Myeloid Leukemia	1	0.05	18.21	(0.46, 101.45)	-	-	-	-	1	0.05	19.61	(0.50, 109.29)
Other Leukemia	2	0.05	36.36*	(4.40, 131.36)	1	0.01	77.25*	(1.96, 430.43)	1	0.04	23.78	(0.60, 132.49)
Aleukemic, Subleukemic and NOS	2	0.05	36.36*	(4.40, 131.36)	1	0.01	77.25*	(1.96, 430.43)	1	0.04	23.78	(0.60, 132.49)
**Kaposi Sarcoma**	3	0.67	4.46	(0.92, 13.04)	1	0.29	3.39	(0.09, 18.92)	2	0.38	5.29	(0.64, 19.13)
**Miscellaneous**	2	1.42	1.41	(0.17, 5.08)	-	-	-	-	2	1.27	1.58	(0.19, 5.70)

Individual cancer sites not included had 0 observed cases

IR-solid tumors defined as those solid tumors with standardized incidence ratios >1
